# Biological Effects of *Spirulina* (*Arthrospira*) Biopolymers and Biomass in the Development of Nanostructured Scaffolds

**DOI:** 10.1155/2014/762705

**Published:** 2014-07-23

**Authors:** Michele Greque de Morais, Bruna da Silva Vaz, Etiele Greque de Morais, Jorge Alberto Vieira Costa

**Affiliations:** ^1^Laboratory of Microbiology and Biochemical, College of Chemistry and Food Engineering, Federal University of Rio Grande, P.O. Box 474, Avenida Itália, Km 8, 96203-900 Rio Grande, RS, Brazil; ^2^Laboratory of Biochemical Engineering, College of Chemistry and Food Engineering, Federal University of Rio Grande, P.O. Box 474, 96203-900 Rio Grande, RS, Brazil

## Abstract

*Spirulina* is produced from pure cultures of the photosynthetic prokaryotic cyanobacteria *Arthrospira*. For many years research centers throughout the world have studied its application in various scientific fields, especially in foods and medicine. The biomass produced from *Spirulina* cultivation contains a variety of biocompounds, including biopeptides, biopolymers, carbohydrates, essential fatty acids, minerals, oligoelements, and sterols. Some of these compounds are bioactive and have anti-inflammatory, antibacterial, antioxidant, and antifungal properties. These compounds can be used in tissue engineering, the interdisciplinary field that combines techniques from cell science, engineering, and materials science and which has grown in importance over the past few decades. *Spirulina* biomass can be used to produce polyhydroxyalkanoates (PHAs), biopolymers that can substitute synthetic polymers in the construction of engineered extracellular matrices (scaffolds) for use in tissue cultures or bioactive molecule construction. This review describes the development of nanostructured scaffolds based on biopolymers extracted from microalgae and biomass from *Spirulina* production. These scaffolds have the potential to encourage cell growth while reducing the risk of organ or tissue rejection.

## 1. Introduction

Tissue engineering, the interdisciplinary field that combines techniques from cell science, engineering, and materials science, has the potential to reconstitute damaged tissues and organs using cells that are supported on scaffolds where the components of the extracellular matrix can segregate during tissue and organ formation. It is important to choose a scaffold material that stimulates cells to produce structures [1]; therefore, much research has been carried out on natural organic materials. The natural compounds that are incorporated into the scaffolds can act as a substratum for cellular growth by stimulating cell growth and anchoring with lower risk of tissue rejection when compared with synthetic sources.

*Spirulina* is a prokaryotic microalga, order Cyanophyceae, division Cyanophyta (Cyanobacteria). It has a distinctive arrangement of multicellular cylindrical trichomes in an open helix throughout its length. The helical shape of the trichomes is characteristic of the genus, but the length and size of the helix vary with species [2]. In 1981, the Food and Drug Administration (FDA) declared “*Spirulina* is source of protein and contains several vitamins and minerals. It can be legally marketed as a food or a food supplement if it is precisely defined and free from contaminants and adulterants” and is categorized by the FDA as “Generally Recognized as Safe” (GRAS) [3].

*Spirulina* biomass stimulates important biological processes and exhibits antiallergenic, antibacterial, antifungal, anti-inflammatory, antioxidant, and immunomodulating properties [4]. Thus,* Spirulina* LEB 18 biomass incorporated into scaffolds stimulates cell growth and tissue regeneration [5–7].

Nanofiber scaffolds have the potential to be used in tissue engineering because they can reproduce the structure and function of the native extracellular matrix [8]. Electrospun scaffolds have attracted attention because of their characteristics: they have a high surface area in relation to fiber diameter, a high porosity that stimulates cell growth and connections between cells, and good nutrient diffusion and they encourage angiogenesis/vascularization during tissue regeneration [9].

The synthetic polymers normally used to produce nanofiber scaffolds can be replaced by* Spirulina* biopolymers, which are biodegradable and biocompatible with cells and tissues [5].* Spirulina* biomass can be added to the polymer solutions used in nanofiber production to produce scaffolds that incorporate* Spirulina's* properties; this is possible because electrospinning does not involve extreme temperatures or pH that would reduce the biological activity of the biomass or its nutrients. Depending on the solvent used to prepare the polymer, the internal components (proteins, fatty acids, and biopolymers) of the biomass can be made available within the scaffolds to stimulate cells or tissues [4].

Polyhydroxyalkanoates (PHAs), a family of biopolymers that includes polyhydroxybutyrate (PHB), can be extracted from various microorganisms, including* Spirulina*, and used to provide atoxic biocompatible scaffolding for human tissue and organ culture. Low molecular weight PHB has been detected bound to human serum albumin and low-density lipoproteins. It degrades into (R)-*β*-hydroxybutyric acid, a naturally occurring mammalian metabolite present at serum concentrations of 3 mg dL^−1^ to 10 mg dL^−1^ in adult humans and presents no health risks. The fact that PHB degrades into such atoxic compounds may explain its biocompatibility with cultured cells and tissues. Not only do* Spirulina* biopolymer nanofiber scaffolds have a lower risk of rejection in human tissue culture but they also contain advantageous bioactive compounds that are present in the* Spirulina* biomass [10, 11].

This review describes the progress made in tissue engineering when* Spirulina* biomass and biopolymers are used in the production of nanostructured scaffolds that promote cell growth while decreasing the risk of tissue and organ rejection.

## 2. Tissue Engineering

Since its beginnings, this field has focused on the development of biological substitutes for the recuperation, regeneration, or substitution of defective tissues [12]. Permanent implants often result in chronic inflammation, which can lead to severe clinical complications. Implants developed using biomaterials could be a viable alternative to reestablish the normal functions of damaged tissues and organs [13].

The process of using tissue engineering to restore or substitute tissues or organs damaged by accidents, congenital defects, or diseases involves the* in vitro* propagation of viable cells attached to biological or synthetic supports, known as scaffolds [6]. After cellular cultivation, the scaffold is implanted into the patient and degrades when the new organ or tissue is formed [13–15].

### 2.1. Scaffolds

Scaffolds are three-dimensional structures that guide tissue development* in situ* at the site of interest depending only on the growth of the surrounding tissue [16]. The scaffold should be selected according to the type of repair and the tissues or organs to be reconstituted, and the scaffold surface is selected according to the desired interactions between the cells and the scaffold [13, 17].

For effective tissue reconstruction, scaffolds must conform to specific requirements. High porosity and pore interconnectivity are fundamental characteristics for increasing the available specific surface area, which is important not only for cell anchorage and the internal growth of tissues but also for facilitating the distribution and transportation of oxygen, nutrients, and cellular residues [18].

The degradability is a parameter closely related to the solubility of the molds. If the solubility is too high, the scaffolds will be reabsorbed by the body fluids too quickly without accompanying tissue regeneration. However, if the solubility is too low, it will remain for too long in the body and impede regeneration. Therefore, the degradability is associated with the stability of the biomaterial* in vivo* and an appropriate time is extremely important for proper regeneration [6].

The nature of the scaffold's surface can also affect cellular responses that influence the speed of formation and quality of new tissue [17]. The most appropriate scaffold material should be biocompatible and biodegradable, so that it is nonimmunogenic to avoid further surgical intervention when tissue regeneration is complete [19]. Scaffolds are designed to have a cell structure that is similar to the natural ECM and therefore have characteristics that are suitable for cell culture.

## 3. Extracellular Matrix

The extracellular matrix (ECM) is a component of the connective tissue. It is produced by cells and supports the morphological organization and physiological functions that occur during tissue formation [20, 21]. ECM produces the biochemical and biomechanical signals necessary for tissue morphogenesis, differentiation, and homeostasis [22].

Due to their versatile properties, decellularized extracellular matrices have been widely used as a source of biological scaffolds in tissue engineering and regenerative medicine [23–29]. The most important limitations in regenerative medicine are the shortage of autologous tissue and organ donors and the negative immunological responses and pathogen transfer whenever allogeneic or xenogeneic tissues or organs are used [30, 31]. Another advantage of scaffolds obtained from decellularized tissues and organs is the retention of the structure of the original tissue and organs.

Scaffolds made using the electrospinning process mimic the natural extracellular matrix's mechanical and architectural characteristics, enabling the anchoring and migration of cells. Growth factors, drugs, viruses, and proteins can be incorporated in the matrix. The microalga* Spirulina* is a good choice for incorporation in the production of scaffolds [6]. It is important to choose a scaffold material that stimulates cells to produce structures of the correct format and size. Such scaffolds are generally developed from synthetic polymers, which can be incompatible with human cells [1]. Therefore, much research has been carried out on natural organic materials.

### 3.1. Extracellular Matrices Made of Natural or Synthetic Polymers

Polymers are the raw materials for scaffold production in tissue engineering, and several types of biodegradable polymers are utilized in the development of artificial skin, surgical sutures, vascular grafts, bone joining devices, and controlled-release pharmaceuticals [32].

These materials can be classified as natural polymers (including polysaccharides such as alginate, chitin and chitosan, and starch and hyaluronic acid derivatives); proteins (such as collagen, fibrin gel, and soy and silk proteins); synthetic polymers (such as poly(lactic acid) (PLA), poly(glycolic acid) (PGA), and polycaprolactone (PCL)) [1, 33]; and microbial polymers (biopolymers) (such as polyhydroxyalkanoates (PHAs)).

Synthetic polymers are available in potentially unlimited amounts. Their physicochemical properties can be controlled, with their degradation rates and mechanical properties subject to chemical modification [34]. However, many synthetic scaffolds have hydrophobic surfaces that hinder cell recognition by native cells. Natural and microbial polymers are biologically recognized, which makes it easier to reproduce the properties of the tissues to be regenerated, such as their mechanical and cellular anchoring properties [18].

## 4. Nanotechnology and Scaffold Development

Cells interact with their environment via thousands of nanometric interactions. In tissues and organs, cells are located in three-dimensional microenvironments surrounded by other cells and the extracellular matrix. The ECM contains collagen and elastin, which are organized in nanostructures with specific bioactive functions that regulate cellular homeostasis. An essential stage of scaffold development is the creation of synthetic microenvironments that facilitate the formation of a three-dimensional structure to control cell behavior and promote specific cell interactions [35].

Nanotechnology has been used in several biomedical applications, including pharmaceutical transport, biological detection, disease diagnosis, clinical images resolution, and scaffold development [36]. Nanometric tissue engineering can produce biomaterials that regulate the interactions between cells and their microenvironments by the emission of molecular signals [37, 38]. The biometric and physicochemical properties of nanomaterials enable them to stimulate cell growth and regenerate injured tissue [39].

Developing nanofiber scaffolds using the electrospinning process enables the reproduction of the principal extracellular architecture and makes it easy for the cells to unite to tissue because such scaffolds have similar mechanical properties to natural structures [5]. Several authors have studied the application of nanofiber scaffolds in various processes, including the rebuilding of nerves [40] and brain tissue [41], the transport of pharmaceuticals through oral mucosa [42], and the cultivation of stem cells [6].

### 4.1. Production of Nanostructured Scaffolds via Electrospinning

The greatest challenge in the area of tissue engineering is the development of scaffolds that reproduce nanometric tissue architecture. The electrospinning process is the most widely adopted technique for the formation of polymer nanofibers [39], due to the repeatability of this method and the simplicity of scaling it up.

Nanostructured scaffolds obtained via electrospinning have been attracting attention due to their high porosity. They contain interlinked voids that can increase both cellular development and the connections between cells as well as nutrient diffusion, angiogenesis, and vascularization during tissue regeneration [9]. Electrospinning produces nanofibers with a diameter of between 3 nm and 1000 nm and it can be used to process several types of polymers [15, 43].

Electrospinning is carried out by applying a high voltage to a polymer solution in a process that results in nanofiber formation and lengthening due to electrostatic repulsion. The polymer solution is fed at a constant flow rate through a capillary charged with a high voltage (10 kV to 30 kV). When the electric field attains enough energy to overcome surface tension at the tip of the capillary, a “Taylor Cone” forms and the nanofibers are deposited in a stationary or rotating collector where the solvent evaporates and the nanofibers collect [44, 45].

Many parameters influence this process, including the properties of the polymers, the solvent, and the environment. Some of these parameters are viscosity, elasticity, conductivity, solution flow rate, surface tension, capillary diameter, distance between the capillary tip and the collector, polymer concentration, temperature, humidity, and air flow rate [33]. In addition, the manner in which the nanofibers are collected can influence their orientation, with the fibers being deposited either randomly or in alignment [8]. Electrospinning can be accomplished under laboratory conditions to produce a sterile product and can easily be scaled up [46]. Another advantage is the possibility of incorporating growth factors, drugs, viruses, proteins, and other properties into the nanofibers. Thus,* Spirulina* is a good candidate for incorporation into scaffolds [6].

## 5. The* Spirulina* (*Arthrospira*) Microalga

Phytoplankton are aquatic photosynthetic photoautotrophs characterized by the presence of various colored pigments [47]. Photosynthetic phytoplankton include the eukaryotic algae and the prokaryotic cyanobacteria. Biotechnological processes based on phytoplankton have come to the fore due to their potential to produce a wide range of byproducts, including carbohydrates, lipids, minerals, pigments, proteins, and vitamins [48], many of which are natural products, have a high nutritional value, and are commercially important [47].

**Figure 1:**
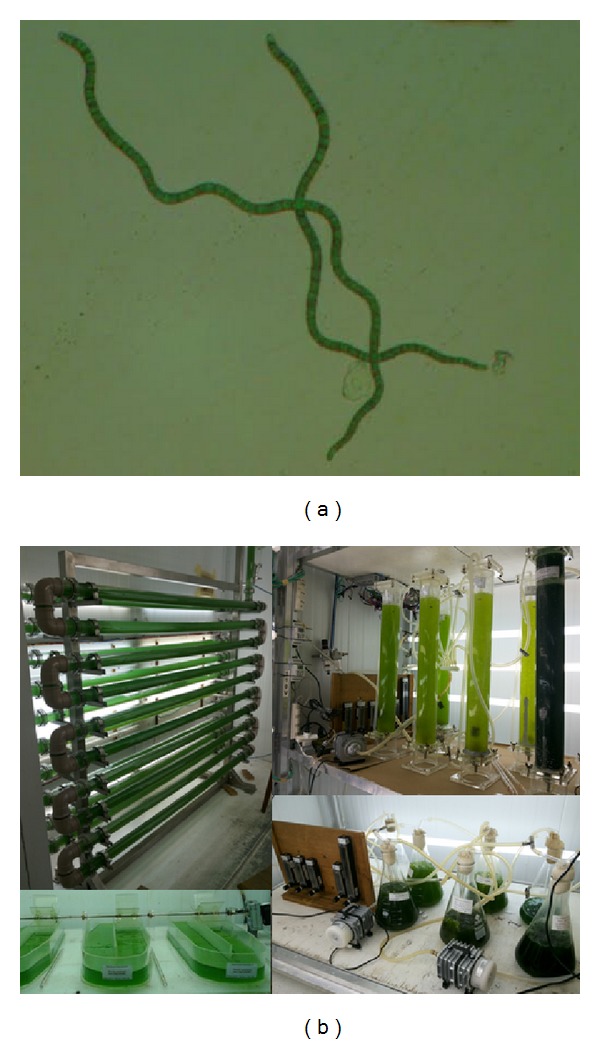
*Spirulina* LEB 18 (a) and culture at the Laboratory of Biochemical Engineering (b).

*Spirulina* is the common name for the product produced from pure cultures of the photosynthetic prokaryotic cyanobacteria* Arthrospira*, the natural habitat of which is alkaline lakes.* Spirulina* was originally given this name due to the spiral nature of its filaments (Figure 1(a)) and was thought to be eukaryotic algae. However, it was later found to be a prokaryotic cyanobacterium belonging to the genus* Arthrospira*. In South America, it has been cultivated as a food supplement since the time of the Aztecs, about 400 years ago [49].

Medical and nutritional studies about* Spirulina* have proliferated since the 1970s, because it is a good source of high quality protein (the concentration of which can reach 70% of the biomass), vitamins (B12 and provitamin A), minerals (especially iron), phenolics, and essential fatty acids [50]. For many years,* Spirulina* has been investigated for application in several fields, especially for use in foods and medicine. It is thus frequently used as a nutritional supplement and is generally regarded as safe when cultivated under conditions of appropriate hygiene. Toxicological studies have demonstrated that* Spirulina* is safe for human consumption.

*Spirulina* has been produced on a large scale in several countries for application as a food supplement and a pharmaceutical product [5]. In Germany (BlueBiotech International GmbH) and the United States (Cyanotech, Eathrise Nutritionals, and Phycobiologics)* Spirulina* is cultivated on a commercial scale for use as dietary supplement [2]. Clinical studies have demonstrated that* Spirulina* biomass has therapeutic properties and that it may be used to treat allergies [51], cancer [52], and HIV [53] and for the reduction of LDL cholesterol [54, 55]. It has also been reported to stimulate the immune system and intestinal lactobacilli, reduce hyperlipidemia and obesity, and counteract the effects of radiation, drugs, and heavy metals [48, 56]. Noninsulin dependent diabetics have shown a reduction in hypoglycemia when* Spirulina* was added to their diet [55].

Since 1996, the Laboratory of Biochemical Engineering (LEB) of the Federal University of Rio Grande (FURG), Brazil, has been running a research program on the cultivation of microalgae and other phytoplankton (Figure 1(b)).

### 5.1. *Spirulina* LEB 18 Biopolymers Used in Nanostructured Scaffolds

The first step in tissue reconstruction consists of the selection of the support material for the cells. During this phase, consideration must be made regarding the type of lesion being repaired along with its location in the body and the extension of the lesion. Permanent implants can cause inflammation, which, although a normal response to a foreign body, can result in more severe clinical complications such as tissue contraction [13].

Collagen-based scaffolds are currently substituted by supports produced from biodegradable polymers [57]. Biodegradable microbial polymers have potential application in the formation of nanostructured scaffolds. Such polymers include the polyhydroxyalkanoates (PHAs), a group of about 150 polymers that has been attracting medical interest. This group includes polyhydroxybutyrate (PHB), poly(3-hydroxybutyrate) (P3HB) and its copolymers, poly(4-hydroxybutyrate) (P4HB), 3-hydroxyvalerate (PHBV), and 3-hydroxyhexanoate (PHBHHx) [58]. The most studied polymer of the group is PHB, which is biodegradable, thermoplastic, and easily processed, making it a good candidate for the development of biodegradable scaffolds [59].

Human biocompatibility is one of the advantages of biopolymers compared with synthetic biodegradable polymers, and they can be used to produce scaffolds that facilitate the anchoring of implanted cells to the tissue that is being regenerated [60]. The United States Food and Drug Administration (FDA) has approved PHB for food packaging. Furthermore, because PHB is biocompatible with cells and tissues and is easily absorbed by the human body it can be used in the medical-pharmaceutical field for sutures, bone prostheses, cardiovascular grafts, orthopedic pins, and implants, as well as in tissue regeneration and repair [61]. Formulations of PHB biopolymer can be used as a matrix for the development of controlled-release medications such as hormones and other pharmaceuticals. The sodium salt of PHB can also be used as an anesthetic [62]. Neural stem cells have been produced in PHB scaffolds and have the potential to repair central nervous system lesions. PHB can also be used to regenerate bones, cartilage [58], and nervous and cardiovascular tissues [63].

PHAs are biodegradable and therefore they do not produce any toxic substances during their metabolism. In the environment, bacteria and fungi secrete an extracellular depolymerase that readily degrades polymeric PHAs into their monomers. In mammalian tissues, degradation products are absorbed through the cellular wall and metabolized [64]. The degradation rate of PHAs depends on many factors. Some factors, such as temperature, humidity, pH, and nutrient supply, are related to the environment, while other factors, such as additives composition, crystallinity, and surface area, are intrinsic to the biopolymer [65].

During PHB synthesis, two acetyl-CoA molecules are joined in a condensation reaction catalyzed by the enzyme 3-(*β*-ketothiolase) to form acetoacetyl-CoA. This enzyme competes for acetyl-CoA with several other metabolic pathways, such as acetate and citrate formation and fatty acid synthesis [66].

The product is reduced to 3-hydroxybutyryl-CoA in a reaction catalyzed by acetoacetyl NADPH-dependent reductase. High concentrations of NADPH and NADH inhibit the enzyme citrate synthase, which is responsible for feeding acetyl-CoA into the tricarboxylic (TCA) cycle, making acetyl-CoA available to 3-*β*-ketothiolase and enabling PHB to be synthesized by the polymerisation of 3-hydroxybutyryl-CoA units of by PHA synthase. The biosynthesis of PHB-HV proceeds with precursors such as acetic, itaconic, propionic, oleic, or valeric acid. Propionic acid is the valerate precursor that is most commonly employed in PHB-HV biosynthesis [66].

Polyhydroxybutyrate can be produced by prokaryotic microorganisms such as* Spirulina*, where it functions as carbon and energy reserve [67].

**Figure 2:**
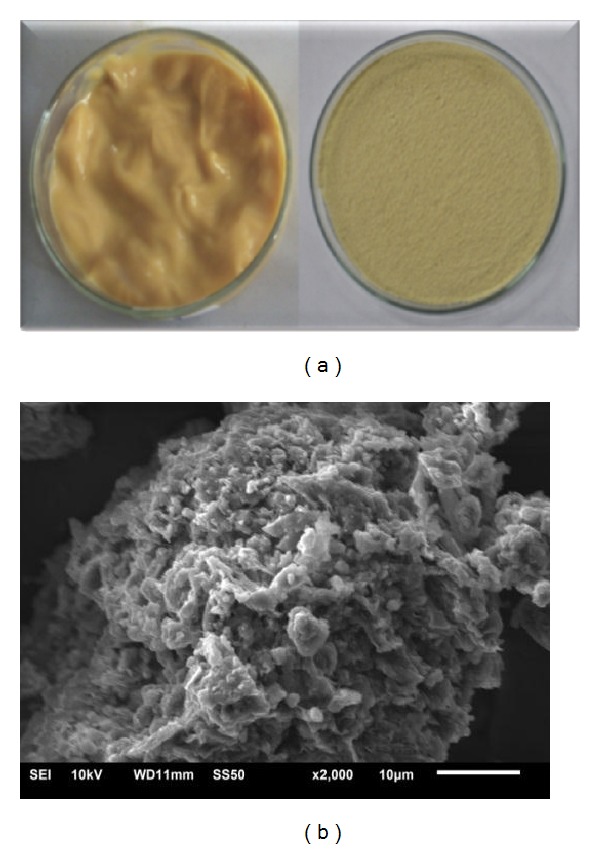
Polyhydroxybutyrate biopolymer produced from the biomass of* Spirulina* strain LEB 18. Biopolymer before and after drying (a) and scanning electron microscopy of the surface of PHB with 2,000x magnification (b).

Since 2007, our team has studied PHB from microalgae. These studies investigated different genera and species of microalgae that produce this biopolymer, as well as physicochemical characterization (scanning electron microscopy, gas chromatography, thermal analysis, differential scanning calorimetry, color, and opacity), optimization of the extraction/purification process, and applications of the biopolymer in the development of biofilms, nanofibers, and nanocapsules (Figure 2).

### 5.2. Physicochemical and Biological Properties and Stem Cell Cultivation of the Scaffolds Made with the Incorporation of* Spirulina* (*Arthrospira*) Biomass or Biopolymers Obtained from the Microalgal Biomass

Since 2007, our team has studied the development of nanofibers produced from PLA, polyethylene oxide (PEO), and PHB extracted from LEB 18 and the incorporation of LEB 18 biomass or some of its metabolites (such as C-phycocyanin).

**Figure 3:**
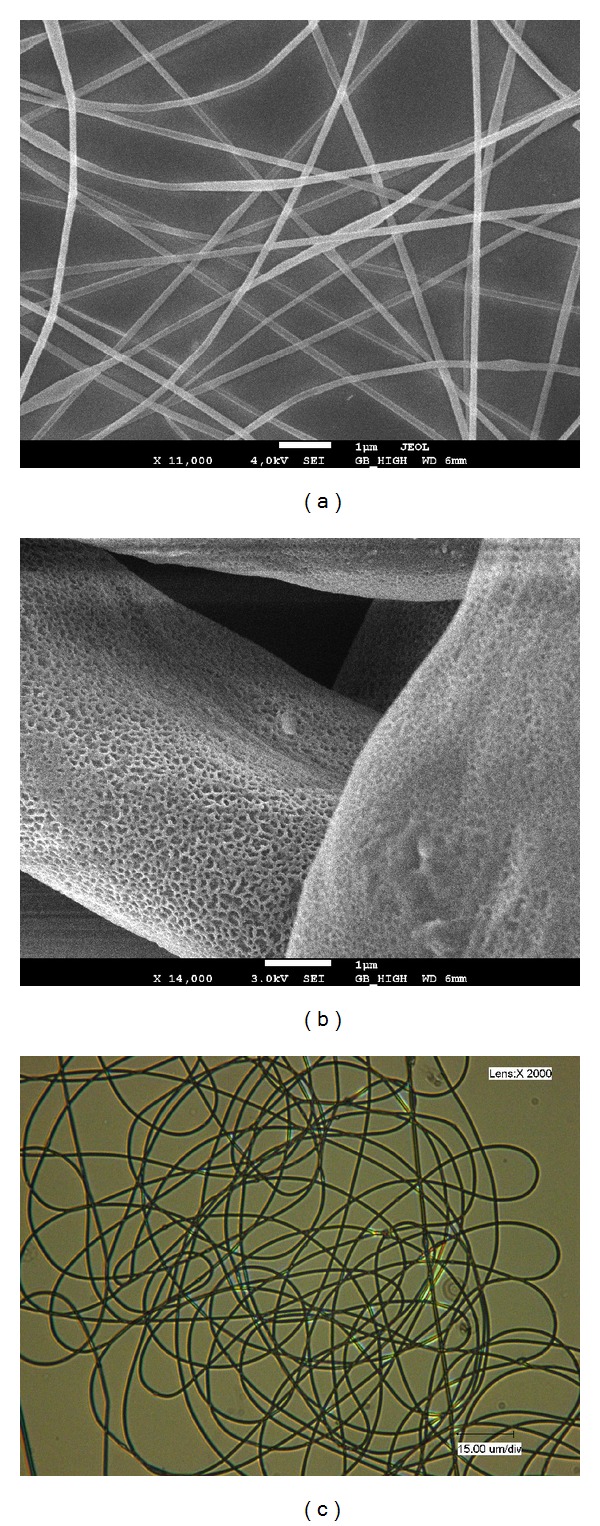
Nanofibers produced with 65% polyethylene oxide and 35%* Spirulina* LEB 18 biomass (a), nanofibers incorporating 25% LEB 18 polyhydroxybutyrate and 5% LEB 18 biomass (b), and optical image of PHB nanofibers incorporating* Spirulina* LEB 18 biomass. 2,000x magnification (c).

The formation of nanofibers via electrospinning is dependent upon the properties of the solution used and the electrical set-up. Morais et al. [5] observed that the addition of* Spirulina* LEB 18 biomass to nanofibers results in a strong increase in conductivity. Nanofibers with* Spirulina* LEB 18 biomass free of beads were produced with diameters of 107 nm (Figure 3(a)) [5].

The elasticity, tensile strength, and breaking elongation of* Spirulina* LEB 18 PHB nanofibers were higher than those of commercial PHB samples. The general finding is that nanofibers composed of* Spirulina* LEB 18 PHB have surprisingly enhanced mechanical properties when compared with nanofibers composed of commercial PHB. These nanofibers had high porosity, and the cells filled the matrix structure, thus enabling the arrival of nutrients and growth factors and removal of metabolic products (data not published) (Figure 3(b)) [68].

The PHB extracted from* Spirulina* and commercial PHB-HV5 and PHB-HV12 were electrospun with and without the addition of sodium chloride or LEB 18 biomass. Electrospinning of 22% w/w* Spirulina* PHB without the addition of sodium chloride or LEB 18 biomass produced uniform nanofibers with a diameter of about 750 nm, while the addition of sodium chloride reduced the diameter to about 480 nm, and the addition of 5% w/w LEB 18 biomass reduced it to about 310 nm. It is important to note that if biomass is added to the spinning solution, PHB nanofibers can be spun with PHB concentrations as low as 7% w/w.

This produces nanofibers with markedly reduced fiber diameters, which could be of importance for membranes produced from such nanofibers. One reason for the reduced nanofiber diameter in the presence of LEB 18 biomass may be that the biomass also contained some PHB, although this cannot be the main reason because the amount of biomass was small. This phenomenon needs further research. Spinning nanofibers using lower concentrations of PHB would reduce production costs.

In another study carried out by our team, all of the electrospinning conditions used for the development of nanofibers with PHB extracted from* Spirulina* sp. LEB 18 were tested for commercial PHB. The commercial biopolymer did not form fibers under any of the conditions, forming only drops, while the PHB extracted from LEB 18 produced nanofibers with a diameter of 470.1 nm. The conditions that formed the smallest diameters were PHB polymer solution extracted from LEB 18 with a concentration of 20% (w/v), flow rate of 150 *μ*L*·*h^−1^, capillary diameter of 0.45 mm, and voltage of 24.1 kV.

The addition of LEB 18 biomass can provide additional optical functionalization of the nanofibers and affect the transmission of light because it produces a nanofiber with a strong green color (Figure 3(c)).

In recent studies where nanofibers incorporating phycocyanin were developed, resistance to the thermal degradation of this biopigment increased when compared with the phycocyanin alone. This showed that the nanofibers produced via electrospinning may protect the added bioactive compounds (data not published) [69].

Scaffolds of poly-D,L-lactic acid (PDLLA) associated (or not) with* Spirulina* LEB 18 biomass (PDLLA/Sp) were developed with the aim of closely mimicking the natural ECM [6]. This resulted in nanofibers ranging from 163 to 581 nm in the PDLLA matrices and from 91 to 576 nm in the PDLLA/Sp scaffolds [6]. The physicochemical and biological properties of the nanofibers produced with PDLLA/Sp showed that these scaffolds had a high porosity and a large number of interconnected pores. They also had a greater and faster wettability when compared with the PDLLA matrix, and the cells had greater adhesion to PDLLA/Sp scaffolds than to PDLLA alone. The results of the cytotoxic assay showed there was not an increase in cell death. The degradability test showed that the PDLLA/Sp scaffolds had a rapid degradation rate (50% degraded within 60 days). Steffens et al. [6] observed that* Spirulina* LEB 18 biomass was released from the nanofiber while the fiber was being degraded. The authors showed that the PDLLA/Sp was capable of increasing the number of viable cells when compared with scaffolds made of PDLLA alone [6].

In another study, Steffens et al. [70] promoted the cultivation of stem cells with the PDLLA/Sp scaffold produced for testing in an animal model of skin injury. The PDLLA/Sp scaffolds were more moldable and had better adherence to the wound when compared with the PDLLA. The authors observed that PDLLA/Sp was adequate for use in animals because it supported the suture and the mechanical stress, all of the animals survived, and there were no complications related to the procedure [70].

Scaffolds made from 0–25 *μ*g/mL of* Spirulina* nanofibers have been used to produce artificial tissue and* Spirulina* enabled the proliferation of mouse fibroblasts. No cytotoxic effects were encountered [71].

Antibacterial and anti-inflammatory effects are critical when scaffolds are used in humans. This is especially true in patients with serious burns where the external protective skin barrier has been completely lost [6]. The application of 0.1%* Spirulina* extract reduces the levels of the bacteria* Escherichia coli* and* Staphylococcus aureus* to insignificant levels within 30 minutes. A methanolic extract of* Arthrospira platensis* had higher antimicrobial activity than dichloromethane, petroleum ether or ethyl acetate extracts, and volatile antibacterial compounds [7].

*Spirulina* stimulates lymphocytes and other cells involved in the immune response. Phycocyanin, a blue pigment associated with the chlorophyll of this organism, exhibits antioxidant and anti-inflammatory properties (due to the inhibition of the release of histamine) [72].

Biomass containing phycocyanin increased the immunity of mice and stimulated haematopoiesis by affecting the glycoprotein hormone erythropoietin and increasing the production of white blood cells.* Spirulina* C-phycocyanin can eliminate free radicals because it is a cyclooxygenase-2 inhibitor that induces apoptosis in the macrophages of mouse via the activation of lipopolysaccharide- (LPS-) induced macrophage [73].

## 6. Conclusion

The development of nanostructured scaffolds using polyhydroxybutyrate biopolymer and the incorporation of* Spirulina* biomass is a significant advance in the field of tissue engineering. This progress is exemplified by the nanofiber architecture, which reproduces the extracellular matrix while reducing tissue and organ rejection during restructuring because of the biocompatible nature of the matrix. This matrix also stimulates cell growth, better nutrient diffusion, and specific cellular interactions due to the properties of LEB 18 biomass. The use of this technology may result in the development of scaffolds that do not require tissue or organ donors.
